# Analysis of β‐Catenin Signalling Activity Suggests Differential Regulation of Ontogenetically Distinct Dentate Granule Neuron Populations

**DOI:** 10.1002/jdn.70009

**Published:** 2025-02-18

**Authors:** Charlotte Billmann, Iris Schäffner, Jana Heppt, D. Chichung Lie

**Affiliations:** ^1^ Institute for Anatomy Friedrich‐Alexander Universität Erlangen‐Nürnberg Erlangen Germany; ^2^ Institute for Biochemistry Friedrich‐Alexander Universität Erlangen‐Nürnberg Erlangen Germany

**Keywords:** dentate gyrus, neurogenesis, niche, β‐catenin signalling

## Abstract

In mammals, the dentate gyrus of the hippocampus is one of the few regions where neurogenesis continues throughout life. As a result, the dentate gyrus harbours neurons of ontogenetically different origin. Notably, ontogenetically different dentate granule neurons (DGNs) are morphologically distinct and fulfil specialized functions in hippocampal information processing and plasticity. Development of adult‐born DGNs is tightly controlled by signals released by the complex cellular environment of the adult dentate gyrus. In mice, an adult‐like cytoarchitecture of the dentate gyrus is observed only after postnatal Week 2. The question therefore arises when the signalling environment controlling adult neurogenesis is established and whether development of ontogenetically distinct DGNs is subject to the same regulatory pathways. Here, we analyse BATGAL reporter mice to determine the temporal development of β‐catenin‐signalling activity in the murine DGN lineage. We show that the β‐catenin‐signalling pattern, which is essential for precise dendritogenesis and neuronal maturation in adulthood, emerges only around 2 weeks after birth and continues to be refined over the next weeks. These results indicate that the signalling environment controlling adult neurogenesis is only gradually established and suggest that the development of ontogenetically distinct DGNs is controlled by different mechanisms.

## Graphical abstract

Wnt/β‐catenin signalling controls neurogenesis in the dentate gyrus of adult mice. This study describes that the adult pattern of Wnt/β‐catenin signalling in the hippocampal neurogenic lineage is progressively established over the first postnatal weeks.

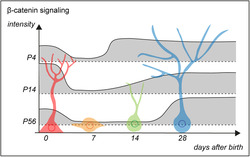

## 1 Introduction

The dentate gyrus serves as the entrance point for cortical inputs into the trisynaptic hippocampal circuitry, which is central for episodic and spatial memory (Amaral 1993). In many mammalian species, dentate gyrus plasticity is supported by the lifelong generation of neurons (Augusto‐Oliveira et al. 2019). This process—termed adult hippocampal neurogenesis—results in the presence of different pools of dentate granule neurons (DGNs): a population generated during the embryonic and/or early postnatal period and an adult‐generated population (Mathews et al. 2010; Spalding et al. 2013).

Adult‐generated DGNs are generated from a quiescent radial‐glia like stem cell (RGL) population in the subgranular zone of the dentate gyrus. RGLs give rise to DGNs through a stereotypic sequence of proliferation, differentiation, and maturation steps. Following cell cycle exit, the newborn neuron enters a maturation process involving extensive changes in morphology, electrophysiological properties and connectivity (Denoth‐Lippuner and Jessberger 2021). A number of these changes take place after the synaptic integration of the neuron into the hippocampal circuitry has been initiated. As a consequence, adult‐generated DGNs are at least transiently distinct with regard to their connectivity and plasticity from their earlier born counterparts (Temprana et al. 2015; Ge et al. 2007; Schmidt‐Hieber, Jonas, and Bischofberger 2004). These differences between the ontogenetically distinct DGN populations are crucial for the function of the dentate gyrus (Tuncdemir, Lacefield, and Hen 2019; Rangel et al. 2013).

Wnt/β‐catenin‐signalling has been identified as a regulatory (Kumamoto et al. 2012; Lie et al. 2005; Kuwabara et al. 2009; Qu et al. 2013; Jang et al. 2013; Seib et al. 2013) or modulatory pathway (Austin et al. 2021) in adult neurogenesis. In mice, timing and dosage of β‐catenin‐signalling regulate the establishment of the stereotypic dendritic morphology and the tempo of dendritogenesis of adult‐born DGNs (Heppt et al. 2020). In the adult neurogenic lineage, canonical Wnt/β‐catenin‐signalling activity follows a ON–OFF–ON pattern (Garbe and Ring 2012; Heppt et al. 2020) with high activity in RGLs, low activity in TBR2+ intermediate precursor cells and immature DCX+ neurons, and reactivation in mature CALBINDIN+ neurons. Functionally, downregulation of β‐catenin‐signalling after the RGL‐stage is essential to establish the basic DGN morphology with a primary dendrite spanning the dentate granule cell layer and initial branching in the inner molecular layer, whereas reactivation of β‐catenin‐signalling is critical for precise refinement of dendritic arbour complexity (Heppt et al. 2020).

Regulatory signals controlling adult neurogenesis are provided by the cellular constituents of the dentate gyrus microenvironment including endothelial cells, microglia, the DGNs themselves and astrocytes (Ming and Song 2011; Matsubara, Matsuda, and Nakashima 2021). In mice, a substantial number of these cell types populate the dentate gyrus only towards the end of the first two postnatal weeks (Schneider et al. 2022; Bond et al. 2020). Likewise, an adult like‐cytoarchitecture of the dentate gyrus subgranular zone—that is, the prospective adult neurogenic niche—is only found from P14 on (Nicola, Fabel, and Kempermann 2015). These observations raise the questions when the regulatory signalling network for adult neurogenesis is established and whether the development of ontogenetically distinct DGN populations is under the control of similar or distinct signals. Here, we begin to investigate these questions by studying when the regulatory ON–OFF–ON pattern of β‐catenin‐signalling is established in the DGN lineage.

## 2 Materials and Methods

### 2.1 Experimental Model and Subject Details

Experiments were carried out in accordance with the European Communities Council Directive (86/609/EEC) and were approved by the government of Middle‐Franconia. Mice were grouped housed in standard cages with ad libitum access to food and water under a 12‐h light/dark cycle. Male and female BATGAL mice (Maretto et al. 2003) were used for experiments. Mice were sacrificed at the age of Postnatal Day (P) 4, P7, P14, P28 and P56. Genotypes were verified by PCR using the forward primer 5′CGG TGA TGG TGC TGC GTT GGA 3′ and reverse primer 5′ACC ACC GCA CGA TAG AGA TTC 3′; conditions were 95°C, 30 s; 59°C, 30 s; and 72°C, 60 s.

### 2.2 Tissue Processing

Mice were anaesthetized using CO2 and transcardially perfused with phosphate‐buffered saline for 5 min at a rate of flow of 20 mL/min followed by perfusion with 4% paraformaldehyde (PFA) in 0.1‐mM phosphate buffer for 5 min. Brains were postfixed in 4% PFA at 4°C for 12 h and subsequently dehydrated in 30% sucrose. Frozen brains were cut at 40‐μm thickness using a sliding microtome (Leica Microsystems).

### 2.3 Immunofluorescent Staining

Sections were rinsed six times in 1× Tris‐buffered saline (TBS) for 10 min at room temperature (RT). Antigen retrieval was performed in 10‐mM Na‐Citrate at 99°C for 10 min and cool down to RT for 25 min. Sections were rinsed three times in MilliQ H_2_O for 5 min, followed by three washes in 1× TBS for 10 min. Slices were incubated in blocking solution (3% donkey serum and 0.25% TritonX‐100 in TBS) at RT for 1 h. Sections were incubated with primary antibodies diluted in blocking solution at 4°C for 72 h. Sections were rinsed six times in 1× TBS for 10 min and incubated overnight with secondary antibody diluted in blocking solution. After four rinses in 1× TBS, sections were incubated in 4′,6‐Diamidin‐2‐phenylindol (DAPI, 1:10,000 in 1× TBS) for 10 min followed by washing in 1× TBS rinsing for 10 min. Sections were mounted on slides with Aqua Poly/Mount.

### 2.4 Antibodies

Primary antibodies:

**Table:** 

Antigen	Host	Manufacturer	RRID	Dilution factor
β‐Galactosidase	Goat	Bio‐Rad	AB_2307350	500
CALBINDIN	Mouse	SWant	AB_10000347	500
DCX	Guinea pig	Merck Millipore	AB_1586992	1000
NESTIN	Mouse	Merck Millipore	AB_94911	500
TBR2	Rabbit	Abcam	AB_778267	500

Secondary antibodies:

**Table:** 

Antigen	Host	Conjugation	Manufacturer	RRID	Dilution factor
Guinea pig IgG	Donkey	Cy3	Jackson ImmunoResearch	AB_2340460	1000
Goat IgG	Donkey	Alexa 488	Thermo Fisher	AB_2534102	1000
Mouse IgG	Donkey	Cy5	Biotium	AB_10853607	1000
Rabbit IgG	Donkey	Cy3	Jackson ImmunoResearch	AB_2307443	1000

### 2.5 Imaging and Quantification

Single‐plane images and z‐stacks for analysis were captured with a Zeiss Axio Observer 7 (Carl Zeiss AG). Setup was performed using the Smart Setup option in ZEN Blue. For representative images and intensity measurements, confocal single plane images and z‐stacks were taken with a Zeiss LSM 780 confocal microscope (Carl Zeiss AG). Detector range and excitation wavelengths were set using the ZEN 2012 Smart Setup option. Standard settings were set to 1024 ×1024 pixels per image and 16‐bit colour depth. Pixel averaging was set to two. Laser intensity, master gain and offset were set individually for every channel to achieve distribution of the fluorescence signal along the dynamical range of the intensity spectrum. Z‐stack step size for coexpression analyses was set to 1.5 μm. Images were processed using Fiji ImageJ. Coexpression analysis were conducted using ImageJ software. For hippocampal region‐specific analysis of reporter activity, > 100 cells per animal and region were analysed from three different animals. For coexpression analysis with cell‐stage specific markers, > 100 cells per animal were analysed from at least three different animals from both hippocampi. Data for the P56 timepoint had previously been published in Heppt et al. (2020).

### 2.6 Fluorescence Intensity Measurements

Fluorescence intensity of the β‐Galactosidase reporter was measured as corrected total cell fluorescence (CTCF) using ImageJ software. To measure nuclear β‐Galactosidase expression levels, the nucleus of a randomly chosen reporter and marker positive cell was outlined. The integrated density, area and mean fluorescence were measured in a single in‐focus plane together with several adjacent background readings. Integrated density of the nuclear β‐Galactosidase signal was corrected by the nuclear area and the mean fluorescence of five background measurements of each staining (CTCF = Integrated Density – [Area of selected cell × Mean fluorescence of background readings]).

### 2.7 Statistical Analysis

For statistical analysis, GraphPad Prism 10.0.0 was used. Statistical significance level α was set to 0.05. Gaussian distribution was tested using the Shapiro–Wilk test. If not applicable, non‐Gaussian distribution was assumed. For analysis of cell counting, ANOVA followed by Tukey's multiple comparisons test was used. The Kruskal–Wallis test followed by Dunn's test for multiple comparisons was used for intensity measurements. Two‐way ANOVA or a mixed model followed by Tukey's multiple comparisons test was used to compare courses of relative mean reporter activity over time. Significances were displayed in GraphPad style with *p < 0.05, **p < 0.01 and ***p < 0.001. Results are represented as mean ± SEM, and the number of individual animals analysed (*n*) is specified for each analysis in the figure legend. For intensity measurements, 100–400 cells were analysed from a minimum of four animals.

## 3 Results

**FIGURE 1:**
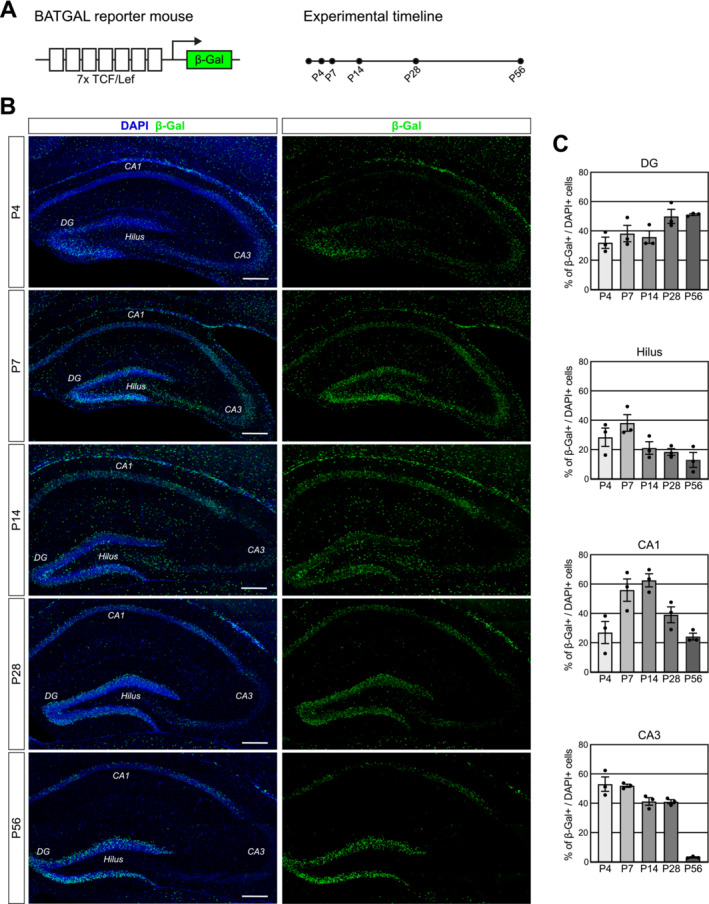
Analysis of global BATGAL reporter expression in the hippocampus from early postnatal stages until young adulthood. (A) Schematic representation of the BATGAL reporter construct and the time points of analysis. (B) Representative images of β‐Galactosidase reporter expression (green) in BATGAL mice on P4, P7, P14, P28 and P56. The nuclear counterstain DAPI is shown in blue. Scale bar = 200 μm. Note that robust reporter expression is observed in the dentate gyrus at all time points. (C) Quantification of the fraction of β‐Galactosidase reporter cells in hippocampal subfields. Dots represent individual animals. *n* = 3 animals per time point.

We sought to determine the pattern of β‐catenin‐signalling activity in mice over the postnatal period and to compare it to the activity pattern in young adult mice. We analysed BATGAL mice (Maretto et al. 2003), in which activity of the Wnt/β‐catenin pathway drives expression of the β‐galactosidase reporter, on P4, P7, P14 and P28, and compared it to our published P56 data (Heppt et al. 2020) (Figure 1A). We observed a dynamic distribution pattern in different hippocampal subfields over time (Figure 1B,C). At P4, high activity was visible throughout the hippocampal formation, including the dentate gyrus blades, the hilus and CA1‐CA3 area. In the dentate gyrus blades and CA1 region, reporter activity appeared constant throughout all timepoints, with the exception of the P14 timepoint, at which activity in the dentate gyrus appeared to be concentrated in the subgranular zone and the outer stratum granulosum at the border to the molecular layer. In contrast to the dentate gyrus and CA1, reporter activity in the hilus and CA3 decreased over time. At P4, numerous cells in the hilus and CA3 expressed the β‐galactosidase reporter. At P14 and P28, the fraction of reporter+ cells was reduced in the hilar region and to a smaller extent in the CA3 subfield. At P56, only sporadic cells with active canonical Wnt at P56 were observed in both regions. In the dentate gyrus, canonical Wnt signalling activity was detectable at each timepoint.

**FIGURE 2:**
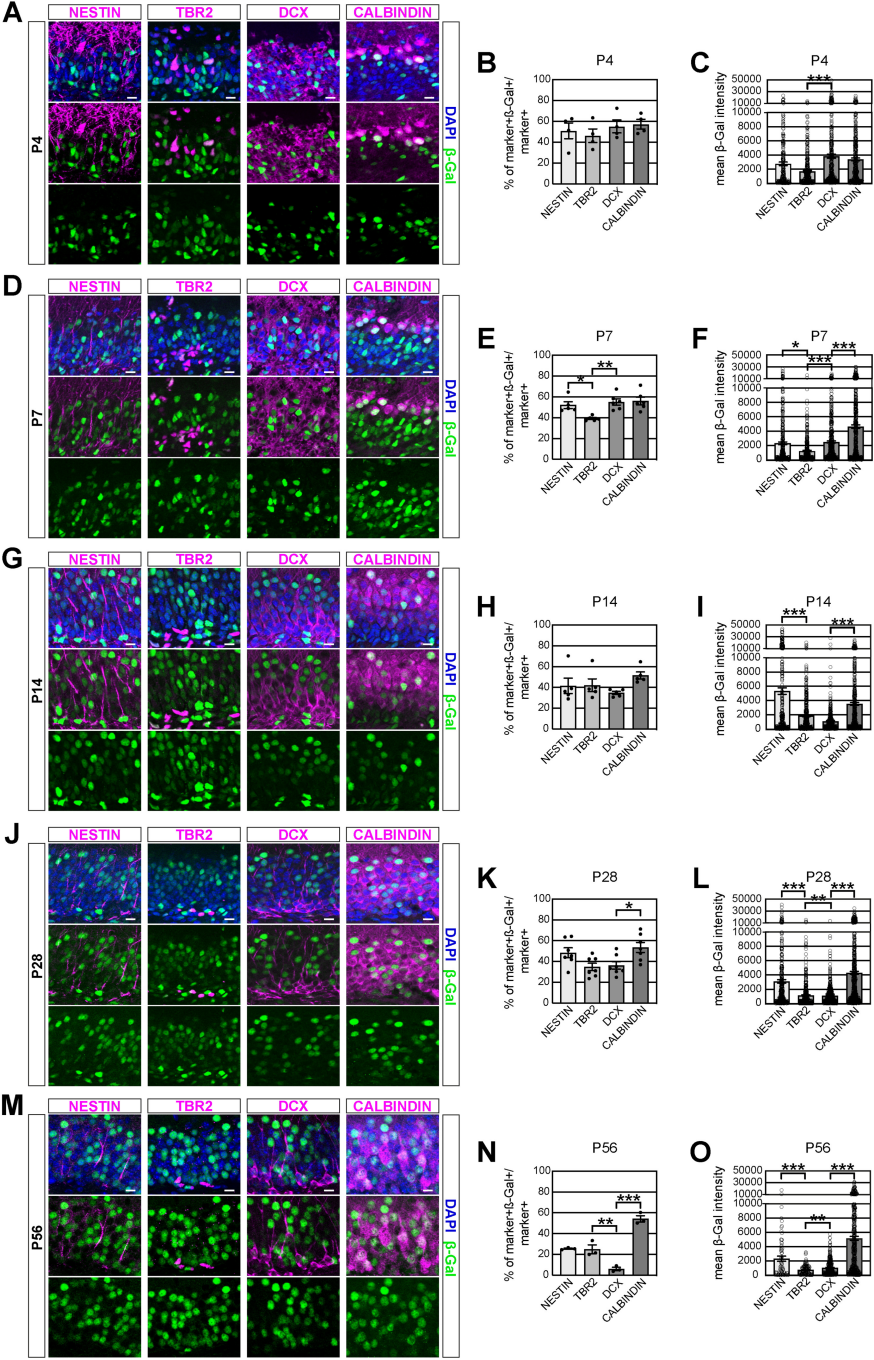
Analysis of BATGAL reporter expression in the dentate granule neuron lineage from early postnatal stages until young adulthood. Left column (A, D, J, G, M): representative images of immunofluorescent stainings for stage‐specific markers (magenta), β‐Galactosidase reporter (green) and DAPI (blue). Middle column (B, E, H, K, N): Quantification of the fraction of reporter+ cells. Dots represent individual animals. P4: *n* = 4, P7: *n* = 6; P14: *n* = 6; P28: *n* = 7; P56: *n* = 3. Right column (C, F, I, L, O): quantification of the mean CTCF in reporter+ cells. Dots represent individual cells (*n* = 100–400 cells). Scale bar = 10 μm. Data represented as mean ± SEM. *p < 0.05, **p < 0.01 and ***p < 0.001.

Next, we investigated β‐catenin‐signalling in the DGN lineage. Reporter activity was determined in NESTIN+ stem cells, TBR2+ intermediate precursor cells (IPCs), DCX+ immature neurons and CALBINDIN+ mature granule neurons and compared between successive stages (Figure 2A–O). On P4, the fraction of reporter+ cells remained stable in the neurogenic lineage (Figure 2B). On P7, we observed a significant decrease in the fraction of reporter+ cells from NESTIN+ stage to the TBR2+ stage (Figure 2E). This decrease was followed by an increase of reporter+ cells amongst DCX+ immature neurons and CALBINDIN+ mature neurons (56% ± 4%). On P14, the fractions of reporter positive cells were comparable across the neurogenic lineage (Figure 2H). An ON–OFF–ON activity pattern was observed on P28 (Figure 2K). Here, TBR2+ IPCs and DCX+ immature neurons showed lower levels of reporter activity than RGLs and mature neurons, respectively. The P28 biphasic activity pattern, however, differed from the activity pattern found in young adult P56 mice, which is in particular characterized by a very low activity in DCX+ immature neurons (Figure 2N) (Heppt et al. 2020).

Next, we used fluorescent intensity measurements of reporter+ cells as a measure to estimate β‐catenin‐signalling activity. On P4, measurements indicated a drop of signalling activity in TBR2+ IPCs, followed by a surge in DCX+ immature neurons (Figure 2C). Notably, at P4, signalling activity appeared comparable between DCX+ immature and CALBINDIN+ mature DGNs. On P7, measurements indicated a significant drop in β‐catenin‐signalling in TBR2+ IPCs, followed by increasing reporter intensities in immature neurons and mature neurons (Figure 2F). P14 and P28 timepoints showed a drop in signalling activity in TBR2+ IPCs and high activity in CALBINDIN+ mature neurons (Figure 2I,L). In contrast to P4 and P7, β‐catenin‐signalling in the DCX+ population seemed to drop with reporter intensities in immature neurons being comparable to (P28) or even lower (P14) than reporter intensities in TBR2+ cells. Hence, from P14 on the pattern of reporter activity increasingly resembled the biphasic ON–OFF–ON activity pattern of the young adult P56 dentate gyrus, which is characterized by low reporter activity in TBR2+ and DCX+ cells (Figure 2O).

**FIGURE 3:**
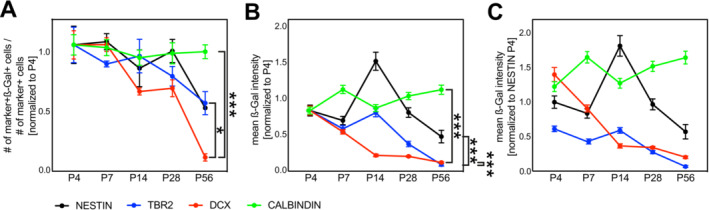
Analysis of cell type‐specific BATGAL reporter expression across time. (A) The fraction of reporter+ cells for each cell type was plotted over time. Values were normalized to the cell‐type specific P4 value, which was set to one. (B) The mean CTCF in reporter+ cells was plotted over time for each cell type. Values were normalized to the cell‐type specific P4 value, which was set to one. (C) The mean CTCF in reporter+ cells was plotted over time for each cell type. Values were normalized to the Nestin P4 value, which was set to one. Data represented as mean ± SEM. *p < 0.05, **p < 0.01 and ***p < 0.001.

The biphasic activity pattern could be consequence of decreasing activity in TBR2+ and DCX+ population or an increasing activity in the NESTIN+ RGL and the CALBINDIN+ mature neuron population. To distinguish these possibilities, we analysed the dynamics of β‐catenin‐signalling for each cell type across the postnatal period (Figure 3). We first considered the fraction of reporter+ cells for each time point as a measure of the trajectory of β‐catenin‐signalling activity (Figure 3A). P4 values were set to one for each cell type. The fraction of reporter+ CALBINDIN+ cells remained constant, whereas all other cell types showed dynamic changes over the postnatal period. The fraction of reporter+ cells amongst NESTIN+ cells was constant until P28 but dropped thereafter by 50%. TBR2+ IPCs showed a similar trajectory. DCX+ immature neurons showed the most pronounced change. On P4 and P7, about 50% of DCX+ cells were reporter+. Reporter positivity decreased to 35% at P14 and P28 and was almost absent from DCX+ immature neurons at P56 (Figure 3A).

We then considered reporter intensities across time as a measure of the development of signalling activity (Figure 3B). P4 values were set to one for each cell type. Reporter intensity in CALBINDIN+ neurons showed little variation from P4 until P56. NESTIN+ cells showed a peak in reporter expression on P14. Reporter intensities in TBR2+ cells decreased after P14 to reach very low levels at P56 (~10% of the P4 value). Reporter expression in DCX+ cells decreased until P14, remained constant between P14 and P28 and dropped after P28 to reach very low levels (~10% of the P4 value) at P56 (Figure 3B).

Finally, we related the CTCF values for each cell type and time point to the CTCF value in P4 NESTIN+ cells for better visualization and comparison of the temporal development of signalling reporter levels between cell types (Figure 3C). This visualization illustrated the dramatic drop in β‐catenin‐signalling strength in DCX+ cells over the postnatal period, as DCX+ immature neurons transformed from being the cell type with the highest reporter levels (compare relative reporter levels between cell types at P4) to being one of the cell types with the lowest reporter levels (compare relative reporter levels between cell types at P56).

## 4 Discussion

In mice, DGNs are born from late embryonic stages until old age. Adult‐born neuron development is paralleled by a biphasic ON–OFF–ON pattern of β‐catenin‐signalling, which controls establishment of the dendritic architecture and the precise timing of dendrite morphogenesis (Arredondo et al. 2020; Heppt et al. 2020; Schafer et al. 2015). Here, we compared β‐catenin‐signalling activity in the DGN lineage from early postnatal stages until young adulthood. A hallmark of β‐catenin‐signalling is the nuclear localization of β‐catenin, which can be reliably documented using biochemical methods. Histological demonstration of nuclear β‐catenin in tissues including the postnatal brain, however, remains challenging and in our hands not reproducible. We therefore analysed BATGAL mice (Maretto et al. 2003), which have been widely used to determine β‐catenin‐signalling activity in multiple contexts. A caveat is the temporal resolution of the β‐Galactosidase reporter. Given its stability, initiation of pathway activity is more reliably detected than inactivation or changes in pathway activity if only scoring cells for being reporter positive or negative. To better account for pathway activities, we not only determined the fraction of reporter+ cells but also compared the intensity of the signal between different cell types.

We find high β‐catenin‐signalling activity in the dentate gyrus throughout all postnatal stages examined, confirming the importance of Wnt/β‐catenin‐signalling for dentate gyrus development (Lee et al. 2000; Galceran et al. 2000; Zhou, Zhao, and Pleasure 2004). However, we observed age‐dependent activity patterns in the DGN lineage. In contrast to the adult dentate gyrus, β‐catenin‐signalling activity is high in IPCs and immature neurons during the first postnatal week and remains substantial until P28. We can only speculate whether and how sustained high β‐catenin‐signalling modulates the development of early‐born DGNs. Dendritogenesis of early‐born neurons requires β‐catenin (Gao et al. 2007). Compared to adult‐born DGNs, dendrite and axon growth of early‐born DGNs is accelerated (Kim et al. 2012; Zhao et al. 2006). Our own work revealed that β‐catenin‐signalling regulates the tempo of dendritogenesis in adult‐born neurons with higher activity accelerating dendrite growth (Heppt et al. 2020). Hence, higher β‐catenin‐signalling activity may be responsible for the higher tempo of dendrite growth of early‐born DGNs.

While accelerating dendrite growth, sustained high β‐catenin‐signalling activity decreases the final dendritic complexity of mature adult‐born dentate granule cells. Interestingly, early‐born DGNs, which encounter the highest levels of β‐catenin‐signalling during maturation (Figure 3B,C), feature lesser dendritic complexity than late‐born neurons (Kerloch et al. 2019). These observations raise the possibility that disparate dendritic morphology amongst ontogenetically distinct DGNs is the result of differences in β‐catenin‐signalling activity during maturation.

How is β‐catenin‐signalling attenuated in adult‐generated TBR2+ IPCs and DCX+ immature neurons but sustained in these cell types during the first postnatal weeks? In the adult neurogenic lineage, attenuation of Wnt/β‐catenin‐signalling activity is achieved by downregulation of expression of signalling components (Schafer et al. 2015). It is possible that this downregulation is adult specific and that expression of β‐catenin‐signalling components is maintained from the RGL to the mature neuron stage during the early postnatal period. Age‐dependent differences in the availability of niche‐derived regulatory signals may also play a role. In adulthood, precursor cells, mature DGNs and astrocytes secrete Wnt‐ligands and inhibitors of Wnt/β‐catenin‐signalling activity to generate an intricate signalling environment controlling adult‐born neuron development (Jang et al. 2013; Lie et al. 2005; Seib et al. 2013; Qu et al. 2010). We find that the adult biphasic ON–OFF–ON pattern is gradually rather than abruptly established and that a pattern, which begins to resemble the adult ON–OFF–ON pattern, emerges on P14. Notably, P14 is the time when the vast majority of DGNs and dentate gyrus astrocytes have been born (Schneider et al. 2022; Bond et al. 2020). Moreover, although cells of the DGN lineage are at early timepoints diffusely spread over the dentate gyrus proper and the hilus, they have assumed an adult‐like distribution in the SGZ at P14 (Nicola, Fabel, and Kempermann 2015). It is therefore tempting to speculate that the emergence of an adult‐like biphasic ON–OFF–ON pattern is initiated by the generation of cellular sources for regulators of the Wnt/β‐catenin‐signalling and the establishment of a distinct spatial organization of the neurogenic lineage to these signalling sources.

Another interesting observation is the trajectory of β‐catenin‐signalling in NESTIN+ cells: We found high activity until P28 with peak activity on P14 and lowest signalling activity in adulthood. Wnt/β‐catenin‐signalling modulates proliferation of NESTIN+ stem cells in adulthood (Qu et al. 2010; Austin et al. 2021; Seib et al. 2013). In contrast to the high proliferative activity of stem cells during early postnatal stages, proliferative activity in adulthood is low with many stem cells being quiescent (Berg et al. 2019). It is possible that the lower β‐catenin‐signalling activity during adulthood is causally linked to the decreased proliferative activity and that decreasing the β‐catenin‐signalling activity induces the entrance of stem cells into quiescence during the early postnatal period (Berg et al. 2019).

## 5 Conclusion

Our data indicate that the Wnt/β‐catenin‐signalling network controlling adult neurogenesis is only gradually established over the first postnatal weeks and that ontogenetically distinct DGNs encounter age‐specific signalling activities during their development. In the future, it will be interesting to understand how the adult neurogenic signalling network is established and whether differences in regulatory signalling pathways underlie the morphological and functional differences of the ontogenetically distinct DGN populations (Kerloch et al. 2019; Lemaire et al. 2012; Masachs et al. 2021; Tronel et al. 2015).

## Author Contributions

*Conceptualization*: Jana Heppt and D. Chichung Lie. *Investigation*: Iris Schäffner and Charlotte Billmann. *Formal analysis*: Charlotte Billmann, Iris Schäffner, Jana Heppt, and D. Chichung Lie. *Resources and funding acquisition*: D. Chichung Lie. *Writing – original draft*: Charlotte Billmann, Iris Schäffner, and D. Chichung Lie. *Writing–review and editing*: Iris Schäffner and D. Chichung Lie. *Supervision*: Jana Heppt and D. Chichung Lie.

## Conflicts of Interest

The authors declare no conflicts of interest.

## Data Availability

The data that support the findings of this study are available from the corresponding author upon reasonable request.
